# Unlocking the adenosine receptor mechanism of the tumour immune microenvironment

**DOI:** 10.3389/fimmu.2024.1434118

**Published:** 2024-06-27

**Authors:** Yecheng Han, Chenshuang Dong, Mingwang Hu, Xinmiao Wang, Guiling Wang

**Affiliations:** ^1^ Independent Researcher, Shenyang, China; ^2^ Key Laboratory of Cell Biology, Department of Cell Biology, Ministry of Public Health and Key Laboratory of Medical Cell Biology, Ministry of Education, China Medical University, Shenyang, China

**Keywords:** tumour microenvironment, adenosine receptor, stromal cell, tumour-associated fibroblasts, immunotherapy

## Abstract

The suppressive tumour microenvironment significantly hinders the efficacy of immunotherapy in treating solid tumors. In this context, stromal cells, such as tumour-associated fibroblasts, undergo changes that include an increase in the number and function of immunosuppressive cells. Adenosine, a factor that promotes tumour growth, is produced from ATP breakdown and is markedly elevated in the tumour microenvironment. It acts through specific binding to adenosine receptors, with A2A and A2B adenosine receptor being primary drivers of immunosuppression. This paper presents the roles of various adenosine receptors in different tumour microenvironments. This review focus on the function of adenosine receptors in the stromal cells and non-cellular components of the tumour microenvironment. Additionally, we summarize and discuss recent advances and potential trends in using adenosine receptor antagonists combined with immunotherapy.

## 1 Introduction

Immunotherapy has become a promising treatment for cancer alongside radiotherapy, achieving significant clinical efficacy. Preclinical studies have demonstrated that immunological approaches, such as immune checkpoint inhibitors (ICIs) and cancer vaccines, can produce effective antitumor responses (1, 2). Additionally, immune checkpoint drugs are currently undergoing clinical trials, yielding encouraging results with reduced adverse reactions. Unfortunately, tumour vaccines are not effective for all tumors, and the complex tumour microenvironment (TME) often renders some solid tumors poorly responsive to ICIs (3). Current research suggests that cytokines secreted by tumour or immune cells within the TME, along with changes in immune cell composition and microenvironmental nutrients, can influence the efficacy of immunotherapy (4, 5). The adenosinergic pathway has long been associated with antitumor immunity (6). Specifically, the use of adenosine receptor antagonists has been demonstrated to enhance the antitumor activity of tumour-infiltration lymphocytes and Natural Killer cells (NK cells) (7, 8). Furthermore, adenosine receptor antagonists can inhibit the immunosuppressive microenvironment, including macrophages infiltration (9). Recent experiments have begun exploring the relationship between adenosine receptors and non-immune components in the TME. Therefore, This artical focuses on summarizing the associations and effects of adenosine receptors with stromal cells and non-cellular components in the TME. A brief overview of recent clinical trials involving adenosine receptor antagonists combined with immunotherapy is also provided. Furthermore, potential combinations to enhance antitumor therapies are considered. This review lays the foundation for studying the relationships and mechanisms between adenosine receptors and TME components, offering insights and strategies for more effective immunotherapy combinations.

## 2 Distribution and mechanism of adenosine receptors

Adenosine receptors belong to the class A family of retinoid-like G protein-coupled receptors. Four members of the adenosine receptor family have been identified: A1 adenosine receptor (A1AR), A2A adenosine receptor (A2AAR), A2B adenosine receptor (A2BAR), and A3 adenosine receptor (A3AR). These receptors, as the natural endogenous receptors for adenosine are involved in numerous physiological and pathological signaling pathways (10). This section summarize the distribution of adenosine receptors under physiological and pathological conditions based on data from the Human Protein Atlas database and relevant literature.

### 2.1 Distribution of adenosine receptors under physiological and pathological conditions

Adenosine receptors A2AAR and A2BAR have been approved by the FDA for drug targeting of G protein-coupled receptors. The *ADORA2A* gene is expressed at the RNA level in various tissues (11, 12), while the A2AAR protein is expressed only in a few tissues, with the highest levels found in the colon, caudate nucleus, and appendix (13, 14). A2AAR protein is also present in the cerebellum, kidney and bone marrow (15). Tissue-specific distribution of A2AAR protein has been observed in endothelial cells of tissues such as the myocardium and kidney. Among immune cells, A2AAR levels are higher in neutrophils and regulatory T cells (Treg cells). Under pathological conditions associated with different tumors, adenosine receptor content varies. In a few cases of ovarian cancer, A2AAR protein expression shows moderate to high positivity in the cytoplasmic and cell membrane. A2AAR protein expression in other cancer tissues is generally negative. The expression of A2BAR protein in various tissues under physiological and tumor conditions has not been fully elucidated. However, tissue-specific distribution has been observed in duct cells, urothelial cells, and keratinocytes related to the urinary system, with high expression levels in immune cells, including bone marrow cells and basophils.

The expression of A1AR protein in various tissues under physiological and tumour conditions is also yet to be fully understood. The *ADORA1* gene is highly expressed, mostly in renal carcinoma. Tissue-specific distribution of A1AR protein can be observed in Muller glial cells, oligodendrocyte precursor cells, oligodendrocytes, and excitatory neurons (16–18). The expression level of A1AR protein in immune cells is not high. A3AR is mainly expressed in macrophages in tissues such as the colon, myocardium and lungs. In contrast, A3AR is highly expressed in eosinophils among immune cells (19).

### 2.2 Mechanisms of adenosine receptors in non-tumour and tumour diseases

This review summarizes the current research status of adenosine receptors and disease, focusing on classical and recent studies. The same adenosine receptor may play different roles in various diseases. A1AR, A2AAR and A2BAR have all been reported to be associated with cardiovascular diseases and diabetic complications, but their roles differ. A1AR is mainly involved in processes such as myocardial hypertrophy, ischemia-reperfusion injury, and various arrhythmias (20, 21). In contrast, A2AAR has been demonstrated to regulate coronary artery dilation (21, 22). Unlike A2AAR, A2BAR is involved in vasoconstrictor effects (21). Furthermore, A1AR is involved in the regulation of gestational hypertension through the HSPA8/β-arrestin1/A1AR axis (23). The potential of A2AAR to treat diseases such as Parkinson’s disease and systemic lupus erythematosus is currently under investigation (24, 25). A2BAR plays a role in schizophrenia and is involved in the regulation of pulmonary fibrosis and alcoholic steatohepatitis (26–28). Compared to other adenosine receptors, A3AR is less well-known and has received less attention in cardiovascular and diabetic diseases. It may, however, be a unique marker and target for inflammatory diseases such as rheumatoid arthritis (29). For more details on specific mechanisms, please refer to **Table 1**. Compared to the other listed adenosine receptors, the mechanisms of A2AAR in tumors are less studied, primarily related to pathways such as mitochondrial oxidative phosphorylation and ERK (37). For further details, please refer to **Table 1**.

**Table 1:** Mechanisms of adenosine receptor in disease.

Adenosine receptors	Condition	Disease	Mechanism	Reference
A1AR	Non-Oncology Diseases	Diabetic nephropathy	Enhanced renal tubular interstitial fibrosis and extracellular matrix deposition observed in A1AR DN mice	(30–33)
		Hypertensive disorders in pregnancy	Regulation through the HSPA8/β-arrestin1/A1AR axis	(23)
		Cardiovascular diseases	A1AR is involved in myocardial hypertrophy, ischaemia-reperfusion injury, various types of arrhythmia, chronic heart failure and arterial hypertension	(20, 21)
		Tauopathies	regulation or inhibition of A1AR modulates the activity of the “ArfGAP with SH3 Domain, Ankyrin Repeat, and PH Domain 1 protein” (ASAP1)	(34)
	Tumour	Breast cancer	Adenosine binding to A1AR down-regulates cAMP concentration, and low cAMP levels stimulate cancer cell growth and proliferation	(35)
		Esophageal cancer	Inhibition of A1AR reduces the proliferation of oesophageal cancer cells, possibly through an increase in cysteinyl asparaginase 3/7 activity	(36)
		Glioblastoma	A1AR had significant anti-proliferative/pro-apoptotic effects on CSC and promoted the differentiation of CSC to a glial phenotype Regulation of ERK/AKT phosphorylation kinetics and hypoxia-inducible factor expression	(37)
A2AAR	Non-Oncology Diseases	Parkinson’s disease	A2AAR forms a heterodimer with dopamine D2 receptors in the CNS and regulates their activity.	(24, 38)
		Cardiovascular regulation	Regulation of vascular endothelial cell and vascular smooth muscle cell-mediated coronary vasodilation Involvement in aortic vasodilation in mice	(21, 22, 39, 40)
		Systemic lupus erythematosus	A2AAR activation reduces the release of pro-inflammatory cytokines and enhances cytokines with anti-inflammatory activity	(25)
		Diabetic retinopathy	A2AAR achieves retinal protection by eliminating inflammation-mediated interactions with the C-Raf/ERK pathway.	(41, 42)
	Tumour	Breast cancer	Expression and role of A2AAR in the hormone-dependent breast cancer cell line MCF-7	(40)
		Melanoma and Lung Cancer	A2AAR activators increase cell proliferation through pathways dependent on PLC, PKCδ, pERK1/2, pJNK and pAKT signaling	(43, 44)
A2BAR	Non-Oncology Diseases	Cardiovascular regulation	A2BAR mediates adenosine-induced vasoconstrictor vasomotor effects in human chorioallantoic membrane vasoconstriction by synthesizing thromboxane receptor activators or related prostaglandin-like agents Blockage of the adenosine A2B receptor prevents cardiac fibroblasts overgrowth in rats with pulmonary arterial hypertension Activation of adenosine A2B receptor alleviates myocardial ischemia-reperfusion injury by inhibiting endoplasmic reticulum stress and restoring autophagy flux A2BAR is involved in altered ventricular function in mice	(21, 22, 45–49)
		Alcoholic hepatitis	A2BAR reduces alcoholic steatohepatitis by upregulating cAMP levels and negatively regulating the NF-kB pathway	(26)
		Idiopathic Pulmonary Fibrosis	Elevated adenosine levels may activate A2BAR, leading to cellular effects associated with the progression of pulmonary fibrosis	(27)
		Diabetic proteinuria	Blocking A2BAR protects against diabetic proteinuria by affecting adhesion plaque kinase activation and adhesion kinetics of podocytes	(50)
		Schizophrenia	Activation of A2B adenosine receptor protects against demyelination in a mouse model of schizophrenia	(28)
	Tumour	Glioblastoma	A2BAR had significant anti-proliferative/pro-apoptotic effects on CSC	(37)
		Breast cancer	A2BAR induces breast CSC cell cycle arrest and apoptosis by down-regulating ERK1/2 cascade response	(51, 52)
		Ovarian cancer	A2BAR agonists induce apoptosis through the mitochondrial signaling pathway	(53)
		Glioblastoma	Targeting A2BAR is an effective treatment for GBM recurrence	(54)
		Lung cancer	A2BAR as one of the receptors involved in the regulation of the EMT process	(55)
		colorectal cancer	A2BAR antagonists alter the cellular redox state and increase basal oxygen consumption rate and mitochondrial oxidative phosphorylation	(56, 57)
A3AR	Non-Oncology Diseases	Neurotoxicity	Prevention and reversal of chemotherapy-induced neurotoxicity in mice	(58)
		Rheumatoid arthritis	A3AR is a promising therapeutic target for rheumatoid arthritis	(29)
		Reversing Pain	A3AR agonists reverse neuropathic pain through T cell-mediated IL-10 production	(59, 60)
		Osteoarthritis	A3AR activation attenuates osteoarthritis progression by inhibiting NLRP3/caspase-1/GSDMD-induced signaling	(61)
		Plaque psoriasis	A3AR Agonist Piclidenoson Shows Increased Response to Psoriasis Treatment Over Time and a Good Safety Profile	(62)
	Tumour	Most of the cancers	A3AR activation also results in the inhibition of PI3K/Akt and a subsequent deregulation of nuclear factor κB (NF-κB) and MAPK signaling pathways resulting in anti-inflammatory and anticancer effects	(32, 33)
		Hepatocellular carcinoma	A3AR agonists induce dysregulation of Wnt and NF-kB signaling pathways, leading to apoptosis in HCC cells. Tumor cell proliferation is reduced, with decreased accumulation of G1 phase cells and inhibition of DNA and RNA synthesis. Reduced protein expression levels of β-conjugated proteins, patched1 (Ptch1), and glioma-associated oncogene homologous zinc finger protein 1 (Gli1) were found. The protein expression levels of cell cycle protein D1 and c-Myc were reduced.	(63, 64)
		Lung cancer	Doxorubicin and A3AR agonist therapies have lowered TNF-alpha levels	(65)
		Breast cancer	A3AR agonists induce cell cycle arrest and apoptosis in BCSCs by inhibiting ERK1/2 and GLI-1 cascade responses	(66)
		prostate carcinoma	Reduced protein expression levels of PKAc and elevated levels of GSK-3 β protein lead to β-catenin destabilization, which inhibits cell cycle protein D1 and c-myc expression	(67)
		Pancreatic cancer	Tumor cell proliferation is reduced, with decreased accumulation of G1 phase cells and inhibition of DNA and RNA synthesis. Reduced protein expression levels of β-conjugated proteins, patched1 (Ptch1), and glioma-associated oncogene homologous zinc finger protein 1 (Gli1) were found. The protein expression levels of cell cycle protein D1 and c-Myc were reduced.	(64, 68)

### 2.3 Effect of adenosine receptors on components of the TME

The TME is a highly structured ecosystem composed of various immune cells, tumor-associated fibroblasts (CAFs), endothelial cells (ECs), and extracellular matrix (ECM). The composition of these components may vary depending on the specific tissue type and change with tumor development. The TME promotes tumor progression in a manner conducive to tumor growth and survival. A typical TME is suppressive, characterized by a high number of immunosuppressive cells and an acidic pH environment (69, 70). In the previous section, we described the mechanisms of adenosine receptors in tumor cells. This section will detail the targets and modes of action of adenosine receptors in tumor tissues and organs. The immune microenvironment composition of different tumors is complex and diverse, and the effects of adenosine receptors on these environments vary. **Table 2** provides examples of this.

**Table 2:** Effect of adenosine receptors on components of the TME.

Cancer Type	Adenosine receptors	Microenvironmental components	Phenomenon	Mechanisms of the phenomenon	Reference
Pancreatic	A2BAR	Immune cell	Showed lower abundance of B cells, CD8 T cells, T regulatory cells, NK cells and M2 macrophages		(71)
		Fibrosis and collagen	Promotion of fibrosis and collagen deposition		(71)
Lung cancer	A2BAR	Immune cell	Blocked NK cytotoxic activity and cytokine production	A2BR encounters adenosine on NK cells, and the cyclic adenosine monophosphate (cAMP) pathway is triggered	(72–74)
Most of the cancers	A2AAR	Oxygen	Hypoxia caused by increased adenosine acts by binding A2AAR	Hypoxia-HIF-1α and A2A adenosine receptor-cAMP axis are associated with tumor protection	(75)
Colorectal cancer	A3AR	Cytokine	Increased production of VEGF and Ang-2		(76)
Cervical cancer	A2BAR	Cytokine	Enhancement of IL-10 production		(77)
Melanoma	A2BAR	Cytokine	Inhibition of IL8 and IFN-γ production.		(78, 79)
		Angiogenesis	Enhanced VEGF-α expression and vessel density	Depletion of MDSCs significantly reduced A2B-induced VEGF production	(78, 80)
		Tumour-associated fibroblast	Fibroblasts express altered levels of CXCL12 and FGF2	Altered fibroblast activation protein (FAP) levels in tumors	(81)
	A3AR	Cytokine	VEGF secretion when treated with VP-16 and doxorubicin.		(78)
		Immune cell	A3AR agonist enhances NK cell activity		(82)
Glioblastoma	A2BAR	Angiogenesis	Increased angiogenesis and vascular endothelial growth factor expression	MRS1754 treatment	(54)
		Stem cell	Enhanced migration and invasion of GSCs	Down-regulation of MMP9 activity and expression of epithelial-mesenchymal transition markers	(54)
	A3AR	Angiogenesis	Increased angiogenesis	A_3_AR blockade reduces GSC differentiation to endothelial cell (EC)-mediated angiogenesis under hypoxia.	(83)
	A2AAR	Immune cell	Improvement of T-cell anti-tumour immunity		(84)
Fibrosarcoma	A2AAR	Immune cell	Blocking A2AAR caused CD8 T cells to secrete higher levels of IFNγ	A2AR deficiency improves immunity of endogenous CD8 T cells against MCA205 tumors	(85)
			A2AR adenosine signaling inhibits NK cell maturation		(86)
Breast cancer	A2BAR	Cytokine	Reduced production of IFNγ and perforin		(87)
		Tumour-associated fibroblast	A2BAR affects the TGF-β pathway in CAFs and the phenotype of CAFs	Influencing αSMA in CAFs by AC-PKA-TGFβ	(88)
		Immune cell	Increase in tumour infiltrating CXCR3(+) T cells and decrease in endothelial cell precursors within the tumour		(52)
	A2AAR	Immune cell	A2AAR Changes on T Cells and Natural Killer (NK) Cells	A2AAR Blockade Significantly Enhances IFNγ and Granzyme B Expression via CD8+ T Cells and Enhances PD1 Therapeutic Sensitivity	(7)
			Dendritic cell treatment	Use of A2AAR inhibitors enhances therapeutic potential of DC vaccines	(89)
			Increase in tumour infiltrating CXCR3(+) T cells and decrease in endothelial cell precursors within the tumour		(52)
		Tumour-associated fibroblast	A2AAR affects the TGF-β pathway in CAFs and the phenotype of CAFs	Influencing αSMA in CAFs by AC-PKA-TGFβ	(88)
Osteosarcoma	A2BAR	Cytokine	Reduced production of IFNγ and perforin		(87)
prostate cancer	A2AAR	Immune cell	Increase in tumour infiltrating CXCR3(+) T cells and decrease in endothelial cell precursors within the tumour		(52)
	A2BAR	Immune cell	Increase in tumour infiltrating CXCR3(+) T cells and decrease in endothelial cell precursors within the tumour		(52)

## 3 Mechanisms of adenosine receptors affecting the TME components

### 3.1 Adenosine receptors and immune cells

It is widely believed that immunosuppression leads to resistance to immunotherapy. CD8+ T cells and NK cells are of crucial in killing tumour cells and are also the cells with reduced capacity in the TME. In addition to the reduced lymphocyte activity caused by cytokines secreted by tumors, animal experiments have shown that inhibiting of adenosine receptors can enhance the antitumor capabilities of CD8+ T cells and NK cells (84, 85). Erika et al. demonstrated *in vitro* that *ADORA2B* is a key molecule involved in the CD8+ T cell anti-pancreatic tumour response (71). Simultaneous inhibition of *ADORA2B* and CD73 may enhance CD8+ T cell-mediated antitumor response in patients with pancreatic ductal adenocarcinoma (PDAC) (71). Similarly, inhibition of A2BAR signaling can restore T cell function and proliferation in the breast cancer microenvironment (87). A2AAR agonists have been demonstrated to inhibit the activity of a range of animal and human immune cells, both under healthy conditions and in the presence of tumors (86, 90). The mechanistic changes occurring within different immune cells when various adenosine receptors are antagonized have not been extensively studied. A more comprehensive understanding of these mechanistic changes will aid in developing more effective target designs and drug combinations. future research is expected to fill this knowledge gap.

### 3.2 Adenosine receptors and CAFs

**Figure 1:**
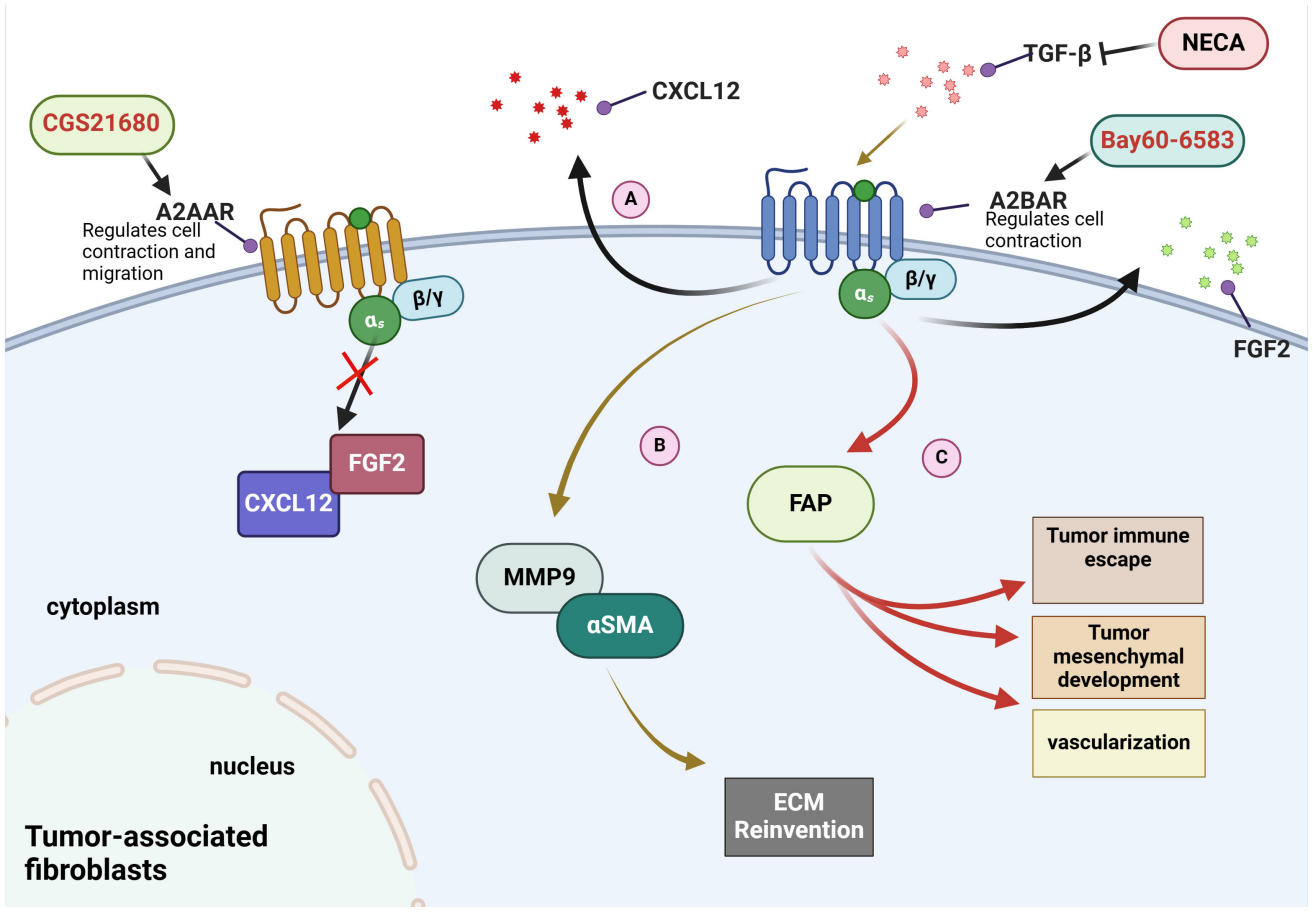
Mechanisms of adenosine receptors on fibroblasts in the tumour microenvironment. **(A)** Bay60-6583, an agonist of A2BAR, has been demonstrated to promote the secretion of CXCL12 and FGF2. **(B)** NECA, an adenosine analogue, inhibits the elevation of MMP9 and αSMA induced by TGF-β in A2BAR-dependent manner. **(C)** A2BAR promotes the expression of FAP to induce immune escape and vascular remodeling.

CAFs play a critical role in tumour development and progression. They provide nutrients such as glutamine, essential for tumour cells when glutamine is depleted in the TME (91, 92). Changes in adenosine receptors, especially A2BAR, are also associated with CAFs. A2BAR is expressed in fibroblasts (93–95). Studies have found that A2BAR inhibitor PSB1115 can reduce the number of CAFs expressing fibroblast activation protein (FAP) and fibroblast growth factor (FGF-2) in the TME of melanoma mice (81). Additionally, the secretion of the cytokine CXCL12 is also reduced (81). The results suggest that MMP9 expression and contractile function in fibroblasts appear to be fully regulated by A2BAR, with no involvement from A2AAR (96). Furthermore, A2BAR can act as a mediator of adenosin- regulated of TGFβ on fibroblasts (96). Please refer to **Figure 1** for specific details.

### 3.3 Adenosine receptors and tumour ECs

**Figure 2:**
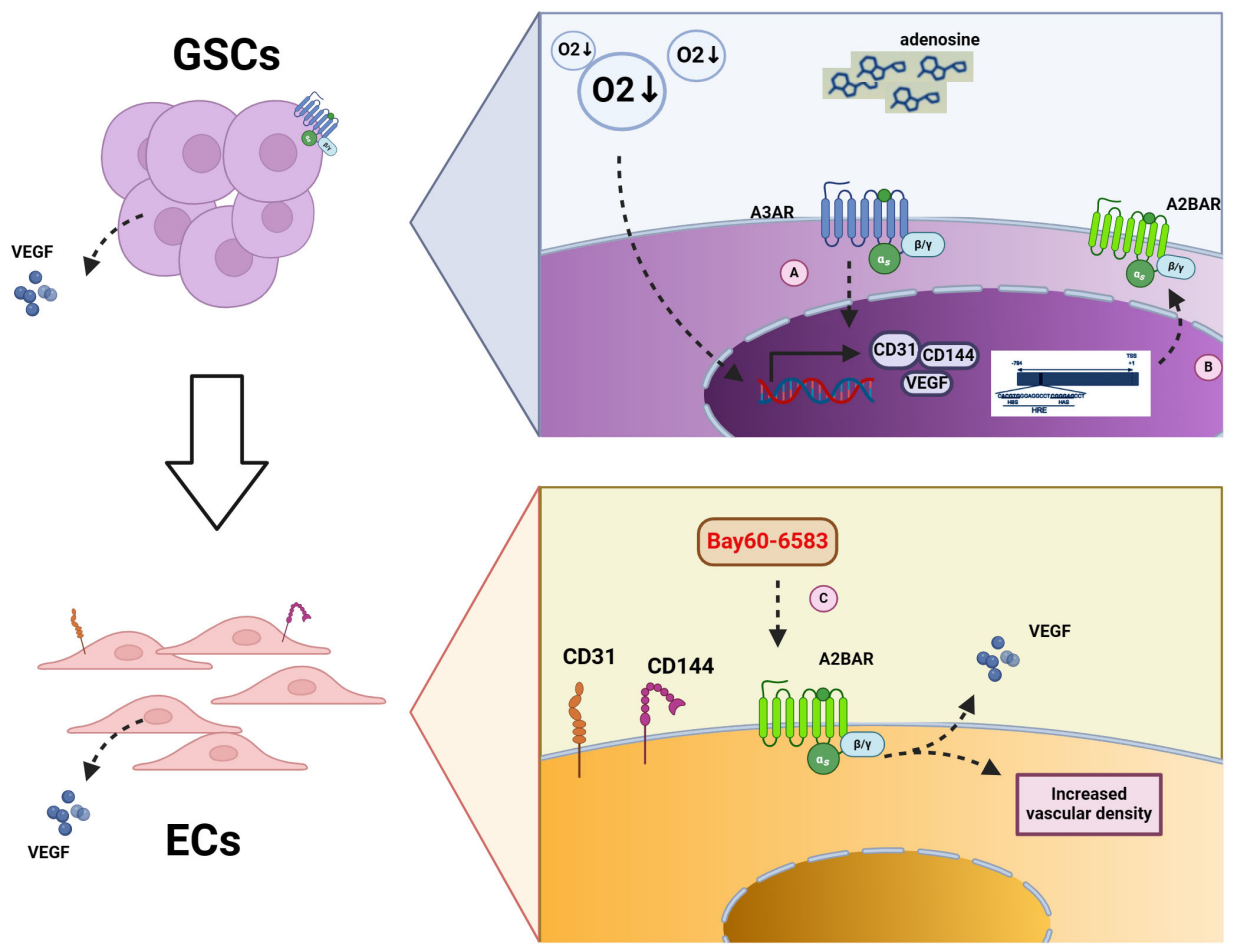
Mechanism of adenosine receptors on GSCs and ECs. **(A)** Increased A3AR content under hypoxic conditions leads to increased transcription of CD31, CD144, and VEGF in GSCs, contributing to the conversion of GSCs to ECs. **(B)** HIFα binds to the promoter of A2BAR, thereby increasing A2BAR content under hypoxic conditions. **(C)** The use of the A2BAR agonist Bay60-6583 results in the increase expression of A2BAR on ECs, which in turn causes the secretion of VEGF and an increase in vascular density.

ECs, also known as vascular endothelial cells, typically refer to the single layer of flat epithelial cells located on the inner surface of the heart, blood vessels, and lymphatic vessels. This layer forms the endothelium (97). The TME is often characterized by hypoxia (91). Studies have shown that adenosine levels increase in the hypoxic TME, and A3AR expression is enhanced on glioblastoma stem -like cells (GSCs). A3AR receptors can promote the production of EC markers, including CD144, CD31, and VEGF, thus promoting the differentiation of GSCs into ECs under hypoxic conditions (83). Another study proposed that the A2BAR promoter contains HIF-1α response element that drive receptor expression in hypoxic cells, including ECs and epithelial cells (52, 98). Furthermore, inhibition of A2BAR on ECs can inhibit tumors under hypoxic TME conditions. A2BAR on ECs has been demonstrated to promote angiogenesis (99). In a mouse melanoma model, the use of the A2B receptor agonist Bay60-6583 increased the expression of VEGF-A and vascular density, thereby promoting melanoma growth (80, 99). For further details, please refer to **Figure 2**.

### 3.4 Adenosine receptors and ECM in TME

ECM plays a critical role in tumour invasion and metastasis. ECM consists of many key components, including collagen, laminin, chondroitin sulfate proteoglycans and hyaluronic acid (100–102). The adenosine-mediated increases in laminin invasion and migration depends on both adenosine receptor-dependent and independent pathways (103). Among adenosine receptors, those regulated by extracellular-5’-nucleotidase (ecto-5’-NT)/CD73 are of particular interest in glioma cell adhesion and tumour cell-ECM interactions (104). Moreover, adenosine receptor A2BAR has been identified as involved in ecto-5’-NT/CD73-mediated arterial calcification (ACDC) (105). High levels of A2BAR in the tumour stroma suggest it may play a significant role. Further research is needed to elucidate the specific adenosine receptors involved in ECM and expand these studies.

### 3.5 Adenosine receptors and mesenchymal stem cells in the TME

Tumors recruit MSCs to the tumour area through the secretion of cytokines, playing a complex role in regulating carcinogenesis and tumourigenesis (106). Subsequently, MSCs can differentiate into more mature MSCs within the tumour area, such as ECs (107). There is little literature on adenosine receptors and MSCs. Studies have shown that elevated adenosine concentrations within tumour tissues can affect the paracrine factors released by MSCs (108). This is related to adenosine receptors, but which specific adenosine receptors are involved remains uncertain and requires further experimental validation.

## 4 Adenosine receptor antagonists and combination therapies

### 4.1 Current status of adenosine receptor antagonists development

Current research is developing both single and dual adenosine receptor antagonists. Zhi Li et al. designed and synthesized several novel A2A/A2B AR dual antagonists centered on triazole-pyrimidine-methylbenzonitrile, with satisfactory test results (109, 110). Compared to clinical antagonist AZD4635, some antagonists performed more prominently *in vitro* immunostimulatory anticancer activity (111). Studies have shown that adenosine receptor antagonists can regulate the immunosuppressive microenvironment, thereby modulating tumour immunity. However, delivering drugs to immune cells remains a challenge. In 2018, it was demonstrated that utilizing CAR-engineered T-cells as active companions, cross-linked multilayered liposome vesicles loaded with A2AAR-specific small-molecule antagonist SCH-58 may be effective. A2AAR-specific small molecule antagonist SCH-58261(SCH) was loaded into 261(SCH) (cMLV) and delivered to tumour-infiltrating T cells deep within the immunosuppressive TME. Adenosine receptor antagonists can be delivered to lymphocytes to exert their effects (112). More precise methods and improvements to existing methods are expected in the future.

### 4.2 Current status of combining adenosine receptor antagonists with other immunotherapies

treating tumors with immunotherapy involves various techniques, including ICIs, is a complex process, encompassing a range of. These include, adoptive cell transfer therapy (ACT), tumour-specific vaccines, small molecule immunotherapeutics and other drugs targeting the TME (23–27). This section discusses the latest progress in combining adenosine receptor inhibitors with immunotherapy. **Table 3** summarizes the combinations of drugs and therapies.

**Table 3:** Clinical trial listing of adenosine receptor antagonists in combination with tumour immunotherapy.

Drugs	Combinations	Clinical trial information		Enrollment	NCT number	Completion date
		Phase	Indications			
Etrumadenant (AB928)	Combination of Etrumadenant, the anti-PD-1 antibody zimberelimab (AB122), AB680 and Sacituzumab govitecan (SG)	1/2	Metastatic castrate resistant prostate cancer (mCRPC)	173	NCT04381832	2024-08
CPI-444(A2AR antagonist)	Combination therapy with ciforadenant, ipilimumab and nivolumab	1/2	Advanced renal cell carcinoma	24	NCT05501054	2026-11-01
PBF-509 (A2AR antagonist)	PBF-509 (A2AAR antagonist) administered as a single agent or in combination with PDR001 (PD-1 antibody)	1	Advanced Non-small Cell Lung Cancer (NSCLC)	92	NCT02403193	2021-11-24
gp96-Ig vaccine	gp96-Ig vaccine in combination with theophylline (A1AR and A2AAR antagonist)	1	NSCLC	36	NCT01799161	2018-04
AB928	Cohort A) etrumadenant + zimberelimab +mFOLFOX-6 +/-bevacizumab vs mFOLFOX-6 +/-bevacizumab Cohort B) etrumadenant + zimberelimab +mFOLFOX-6 +/-bevacizumab vs regorafenib Cohort C) chemotherapy-free combinations of etrumadenant + zimberelimab + other agents	1/2	Metastatic colorectal cancer	227	NCT04660812	2024-07
AZD4635	Module 1: AZD4635 plus durvalumab; Module 2: AZD4635 plus oleclumab.	2	Prostate cancer	59	NCT04089553	2023-04-11
AB928	SRF617 in Combination With AB928 and AB122	2	Prostate cancer	15	NCT05177770	2023-04-05

Data obtained from ClinicalTrials.gov.

The use of ICIs has profoundly impacted the field of clinical adjuvant therapy (113, 114). However, some tumors remain unresponsive to ICIs, prompting scientists to explore combining ICIs with other therapeutic drugs to enhance ICIs efficacy. In recent years, adenosine receptor antagonists have been used in tumour immunotherapy as anticancer small molecule drugs. Takao et al. confirmed that elevated A2AAR expression in metastatic renal cell carcinoma is associated with reduced response and survival rates in patients treated with anti-VEGF drugs and anti-PD-1/anti-CTLA4 antibodies (115). The NCT02403193 trial indicated that PBF-509 (A2AAR antagonist) combined with PDR001 (PD-1 Ab) treatment for NSCLC is safer and more tolerable. Furthermore, a current clinical trial is evaluating the efficacy of combining PD-1 inhibitor zimberelimab with adenosine receptor antagonist etrumadenant. This trial is scheduled to end in August 2024. Another ongoing clinical trial investigates the combination of A2AAR antagonist ciforadenant with CTLA4 blocker ipilimumab and PD-1 inhibitor nivolumab in treating advanced renal cell carcinoma.

In addition to antibodies targeting immune checkpoints, numerous small molecule drugs with immunostimulatory effects are available. These drugs include small molecule inhibitors targeting cell growth and tumour metabolism, VEGF and VEGFR inhibitors, cytokine inhibitors, and more (88, 116, 117). Adenosine receptor antagonists are a special class of small molecule immunotherapeutics. Oral small molecule drugs overcome the limitations of antibody drugs, such as high adverse reactions, long half-life, and inconvenient infusion administration, opening a new field for drug discovery (117). Scientists are studying the effects of combining adenosine receptor antagonists with small molecule immunotherapeutics (used alone or in combination with small molecule immunotherapeutics and ICIs). VEGF is a protein that can stimulates angiogenesis, also inhibits antigen presentation, suppresses T-cell killing, and recruits immunosuppressive cells (117). VEGF antibodies, including ranibizumab and bevacizumab, have been shown to synergistically treat tumors with ICIs *in vitro* and *in vivo* (117, 118). Currently, a trial named NCT04660812 investigates the effects of combining bevacizumab with etrumadenant and zimberelimab. Adenosine is produced by the catalysis of adenosine monophosphate (AMP) by ectoenzyme CD73, making CD73 another important target for therapeutic intervention. Current drugs targeting CD73 include AB680 and OP5244/ORIC-533, as well as antibody drugs (119–121). Exosomes containing CD73 have been demonstrated to inhibit lymphocyte function and to enhance the efficacy of anti-PD-1 drugs (122). AstraZeneca and colleagues have launched clinical trials to study the efficacy of combining oleclumab (CD73 inhibitor) with AZD4635. Similarly, SRF617, a drug targeting surface molecule CD39, has also entered clinical trials. In contrast, the clinical efficacy of combining adenosine receptor antagonists with small molecule immunotherapeutics (cytokines) for tumour treatment has not yet been confirmed.

Tumor-specific vaccines utilize antigenic peptides extracted from tumors to activate immune cells (NK cells and T cells) within the TME (121). For example, heat shock protein GP96 (gp96-Ig) can act as a molecular chaperone, binding intracellular cancer embryonic antigens and progenitor antigens and activating T cells to kill tumour cells. Fariba et al. demonstrated that using nanoparticles loaded with siRNA to inhibit A2AAR and PD-1 immune checkpoints can enhance the efficacy of dendritic cell vaccines (123). Clinical trials conducted by Eckhard et al. demonstrated the efficacy of blocking adenosine receptors with theophylline combined with gp96-Ig tumour vaccine. Further research is deeded to determine the efficacy of combining adenosine receptor antagonists with tumour vaccines.

In addition to immunocellular therapy and checkpoint inhibitors, targeting other components of the tumour stroma, such as CAFs, can also help prevent tumors. In an *in vitro* study of the multitarget combination of the anti-CAFs drug tranilast and anti-tumour drug doxorubicin micelles (DTX-Ms), tranilast alone was less effective in inhibiting tumour growth *in vivo*. Additionally, it could only be used as an adjuvant drug with antitumor drugs (124). Given that adenosine receptor antagonists act on CAFs, it is hypothesized that combining tranilast and adenosine receptor antagonists may have a synergistic effect on CAFs. FAP protein is a core protein of CAFs, and most drugs targeting FAP in clinical trials are PET drugs. Targeting FAP protein radioligands is the latest development in nuclear medicine (125). OMTX705 is the first ADC targeting FAP. This drug is currently being developed for various gastrointestinal tumors (126). Given that A2BAR can regulate FAP protein in CAFs (81), adenosine receptor antagonists have the potential to promote radiodiagnosis or synergize with OMTX705. now *in vivo* or *in vitro* studies have been conducted in this field so far. OMTX705 is currently undergoing Phase I clinical trials (NCT05547321). Moreover, herbal medicines such as epigallocatechin gallate and prunella vulgaris polysaccharide that regulate CAFs may also be associated with various adenosine receptor antagonists in different tumors (127, 128). Further *in vivo* and *in vitro* studies are needed to confirm the link between adenosine receptor antagonists and drugs targeting CAFs.

Drugs can also target cytokines in the TME. These cytokines include IL-10, IL-8, and IFN-γ, which are upregulated in the TME (129–131). Adenosine receptor antagonists can act on the TME through these cytokines (77–79). The combination of adenosine receptor antagonists and cytokine drugs is a promising research field. CXCL8 (3–72)K11R/G31P is an antagonist of CXCL8 and has an inhibitory effect on tumour growth in various tumour types (132–134). So far, no *in vivo* or clinical studies have been conducted on the combination of CXCL8 (3–72)K11R/G31P with adenosine receptors or immunotherapy. An alternative approach is a clinical trial (NCT03400332) evaluating the safety and efficacy of an IL-8 monoclonal antibody (BMS-986253) in combination with the drug Nivolumab, an anti-PD-1 monoclonal antibody. BMS-986253 has been demonstrated to reduce tumour PMN-MDSC and stroma in triple-negative breast cancer. The efficacy of BMS-986253 combined with adenosine receptors or in triple therapy with immune checkpoint therapy remains to be evaluated (135). IFN-γ has complex multifaceted effects in the TME, with its impact on tumour growth depending on the balance between antitumor and protumor effects. Adenosine receptors regulate the secretion of IFN-γ, while IFN-γ acts by binding to adenosine receptors on immune cells such as macrophages (136). Furthermore, no clinical trials have confirmed the synergistic effect of IFN-γ with adenosine receptor antagonists. Additionally, the combined treatment of IFN-γ and adenosine receptors must consider the different microenvironmental conditions of patients, thus requiring extensive experimental validation. The combination of cytokine therapy with adenosine receptor antagonists for tumour treatment is another potential research field in the future.

## 5 Conclusions and prospects

The role of adenosine has been more widely studied both *in vivo* and *in vitro*. Research on adenosine receptors and tumour immunity has primarily focused on the effects of adenosine receptors on immune cells within tumour tissues. However, there is limited research on adenosine receptors and stromal cells or extracellular components in the TME. This review outlines the different roles of adenosine receptors in various TME. The review aims to outline the mechanisms of action between adenosine receptors and stromal cells or extracellular components, laying the groundwork for subsequent development of drugs combining adenosine receptor antagonists with the immune microenvironment. Additionally, this review provides an overview of the current status of combining adenosine receptor antagonist with immunotherapy, focusing on their combination with ICIs, tumour molecular vaccines, and small- molecule immunopharmaceuticals. Furthermore, we present our anticipated vision for the future of combining adenosine receptor antagonists with tumour immune microenvironment therapies (CAFs therapy and cytokine therapy), providing evidence for developing new immunotherapy combinations.

## Author contributions

YH: Conceptualization, Data curation, Software, Validation, Visualization, Writing – original draft, Writing – review & editing. CD: Conceptualization, Data curation, Methodology, Validation, Writing – original draft, Writing – review & editing. MH: Conceptualization, Software, Validation, Visualization, Writing – original draft, Writing – review & editing. XW: Data curation, Methodology, Writing – review & editing. GW: Conceptualization, Funding acquisition, Methodology, Supervision, Writing – review & editing.
