# Correction: Unlocking the adenosine receptor mechanism of the tumour immune microenvironment

**DOI:** 10.3389/fimmu.2026.1786407

**Published:** 2026-02-05

**Authors:** Yecheng Han, Chenshuang Dong, Mingwang Hu, Xinmiao Wang, Guiling Wang

**Affiliations:** 1 Independent Researcher, Shenyang, China; 2 Key Laboratory of Cell Biology, Department of Cell Biology, Ministry of Public Health and Key Laboratory of Medical Cell Biology, Ministry of Education, China Medical University, Shenyang, China

**Keywords:** tumour microenvironment, adenosine receptor, stromal cell, tumour-associated fibroblasts, immunotherapy

## Author affiliation

Affiliation “Independent Researcher, China” was erroneously given as “General Affairs Office of Shenyang Hongqiao Hospital of Traditional Chinese Medicine, Shenyang, China”.

The original version of this article has been updated.

